# Repeat dose NRPT (nicotinamide riboside and pterostilbene) increases NAD^+^ levels in humans safely and sustainably: a randomized, double-blind, placebo-controlled study

**DOI:** 10.1038/s41514-017-0016-9

**Published:** 2017-11-24

**Authors:** Ryan W. Dellinger, Santiago Roel Santos, Mark Morris, Mal Evans, Dan Alminana, Leonard Guarente, Eric Marcotulli

**Affiliations:** 1 Elysium Health, Inc, New York, NY USA; 2 KGK Synergize London, London, ON Canada; 3 Department of Biology, MIT, 77 Massachusetts avenue, 68-280, Cambridge, MA 02139 USA

## Abstract

NRPT is a combination of nicotinamide riboside (NR), a nicotinamide adenine dinucleotide (NAD^+)^ precursor vitamin found in milk, and pterostilbene (PT), a polyphenol found in blueberries. Here, we report this first-in-humans clinical trial designed to assess the safety and efficacy of a repeat dose of NRPT (commercially known as Basis). NRPT was evaluated in a randomized, double-blind, and placebo-controlled study in a population of 120 healthy adults between the ages of 60 and 80 years. The study consisted of three treatment arms: placebo, recommended dose of NRPT (NRPT 1X), and double dose of NRPT (NRPT 2X). All subjects took their blinded supplement daily for eight weeks. Analysis of NAD^+^ in whole blood demonstrated that NRPT significantly increases the concentration of NAD^+^ in a dose-dependent manner. NAD^+^ levels increased by approximately 40% in the NRPT 1X group and approximately 90% in the NRPT 2X group after 4 weeks as compared to placebo and baseline. Furthermore, this significant increase in NAD^+^ levels was sustained throughout the entire 8-week trial. NAD^+^ levels did not increase for the placebo group during the trial. No serious adverse events were reported in this study. This study shows that a repeat dose of NRPT is a safe and effective way to increase NAD^+^ levels sustainably.

## Natural compounds restore NAD+ levels in humans

It’s possible to boost declining levels of NAD^+^, a molecule required for fundamental chemical reactions in the human body, with a dietary supplement. A team led by Leonard Guarente of MIT and Elysium Health gave 120 healthy adult volunteers between the ages of 60 and 80 NRPT (known commercially as Basis) over the course of eight weeks. Volunteers taking the regular dose of NRPT had 40 percent more NAD^+^ compared to their baseline when tested at four weeks and at eight weeks. Others who took a double dose of NRPT had even higher levels of NAD^+^, while those who took the placebo saw no increase. Research shows that NAD^+^ levels decline in animals and humans with age. Boosting NAD^+^ in animals improves age-associated diseases, so researchers now want to understand whether humans, too, may benefit.

## Introduction

The maintenance of efficient cellular metabolism has been shown to play a pivotal role in the prevention of age-associated pathologies and in regulating longevity. Metabolic function is dependent on critical choreography between coenzymes and signal transducing proteins acting as integral metabolism sensors. A prime example is the coenzyme nicotinamide adenine dinucleotide (NAD^+^), and the family of proteins called sirtuins.^1^ NAD^+^ has a canonical role in facilitating hydrogen transfer in key metabolic pathways, such as the conversion of NAD^+^ to NADH for mitochondrial metabolism and subsequent ATP synthesis, which is the energy currency of cells. The sirtuin family of enzymes (SIRT1-7) are NAD^+^-dependent deacylases and key regulators of aging.^2,3^ Beyond acting as a cosubstrate for sirtuins, NAD^+^ is also a cosubstrate for other key enzymes, notably poly (ADP-ribose) polymerases (PARPs), which are involved in DNA repair.^4^ NAD^+^-dependent enzymes are involved in a wide range of activities including DNA damage repair, mitochondrial function, chromosomal integrity, gene expression, epigenetic and posttranslational modifications, and calcium signaling.^5^ Importantly, when NAD^+^ is used as a cosubstrate it is consumed (hydrolyzed), which necessitates constant cellular NAD^+^ biosynthesis through pathways that begin with precursors found in the diet, notably vitamin B3_._ There are several forms of vitamin B3: nicotinic acid (NA), nicotinamide (NAM), nicotinamide mononucleotide (NMN), and nicotinamide riboside (NR). NR has emerged as an efficient vitamin B3 NAD^+^ precursor.^6^ Specifically, when NR was examined head-to-head with NA and NAM in mice, NR exhibited unique pharmacokinetics that resulted in significantly higher peak NAD^+^ concentrations in the liver as well as producing significantly higher levels of several intermediate NAD^+^ precursors including NMN, nicotinic acid mononucleotide (NaMN), and nicotinic acid adenine dinucleotide (NAAD).^7^

Numerous studies have demonstrated that NAD^+^ levels decrease with age,^8,9^ including two human studies that report reduced levels in the skin^10^ and the brain.^11^ As NAD^+^ levels decline with age, NAD^+^ precursors may have significant value in raising such levels and maintaining robust health. The efficacy of NAD^+^ precursors in preventing age-related health problems has been borne out in several recent animal studies.^4,12^ One study demonstrated that mitochondrial dysfunction, a hallmark of aging, was caused by declining NAD^+^ levels in old animals leading to a breakdown of communication between the nucleus and the mitochondria.^13^ Remarkably, one week of NMN administration in old mice was shown to reverse the observed mitochondrial dysfunction in a manner requiring the sirtuin, SIRT1.

In another study, NR was shown to reverse the decline in the number and function of adult stem cells in old wild-type mice.^14^ NR was also shown to significantly increase the lifespan of these animals.^14^ Extension of lifespan and health span by NR was also demonstrated in a mouse with DNA repair defects,^15,16^ including a model of Ataxia Telangiectasia, in which the ATM gene was knocked out.^15^ In these models, NR replenishment of NAD^+^ levels led to improved quality of mitochondria and enhanced DNA repair.

NR treatment has also been shown to boost oxidative metabolism in mice and to protect them from high-fat diet-induced obesity.^17,18^ NR-supplemented mice challenged with a high-fat diet did not gain as much weight as the control mice, and displayed increased oxidation of fatty acids, improved insulin sensitivity and increased energy expenditure. In addition, NR increased mitochondrial biogenesis in muscle tissue and enhanced the endurance performance of these animals. Furthermore, mice supplemented with NR showed increased capacity to maintain body temperature during cold exposure.^17^

NAMPT is the rate-limiting step in the NAD^+^ salvage pathway, which generates NAD^+^ by combining nicotinamide and phosphoribosyl pyrophosphate. Muscle-specific NAMPT knockout mice showed an 85% decline in intramuscular NAD^+^ levels and accelerated age-related muscle degeneration resulting in a loss of strength and endurance.^19^ Similarly, old wild-type mice also showed reduced levels of NAMPT and muscle decline. Administration of NR rapidly ameliorated the aging-triggered muscle degeneration resulting in increased strength and endurance in both the NAMPT knockout and old wild-type mice. Furthermore, NR was shown to be more efficient than NAM in this model.^19^

The first human clinical trial using NR, a crossover study on twelve healthy adults, demonstrated that a single dose of NR significantly increased NAD^+^ levels in blood over a 24-hour period. This study also showed increases in the NAD^+^ metabolome over this time course.^7^ However, the question remained whether increases in NAD^+^ could be sustained over longer time courses by daily administration of NR.

NRPT is a combination of NR and pterostilbene (PT), a naturally occurring analog of the polyphenol resveratrol, which has been found to be a potent SIRT1 activator.^20^ Despite the reported physiological beneficial effect of resveratrol, its bioavailability in humans is poor.^21^ PT exhibits greater bioavailability due to the presence of two methoxy groups that allow it to have increased lipophilic and oral absorption,^22,23^ as well as a longer half-life due to reduced oxidation.^24,25^ Based on these considerations, the combination of NR and pterostilbene is predicted to synergistically support metabolic health through NR providing NAD^+^ to all seven sirtuins and pterostilbene providing additional activation of SIRT1. Sirtuins are known to mediate responses to nutritional and environmental signals including the beneficial health effects of calorie restriction.^4,26,27^ In addition, NAD^+^-dependent activation of sirtuins regulates important physiological processes such as circadian rhythm, glucose and fat metabolism, and normal aging.^2,3^

Therefore, we investigated the safety and tolerability of NRPT as well as its efficacy in sustaining elevated NAD^+^ levels in an initial randomized, double-blind, placebo controlled trial of 120 heathy adults between the ages of 60 and 80. This trial represents the first repeat dose trial for NR as well as the first test of the combination of NR and PT in humans. We report that NRPT increases NAD^+^ levels safely and sustainably.

## Results

### Trial overview

**Fig. 1:**
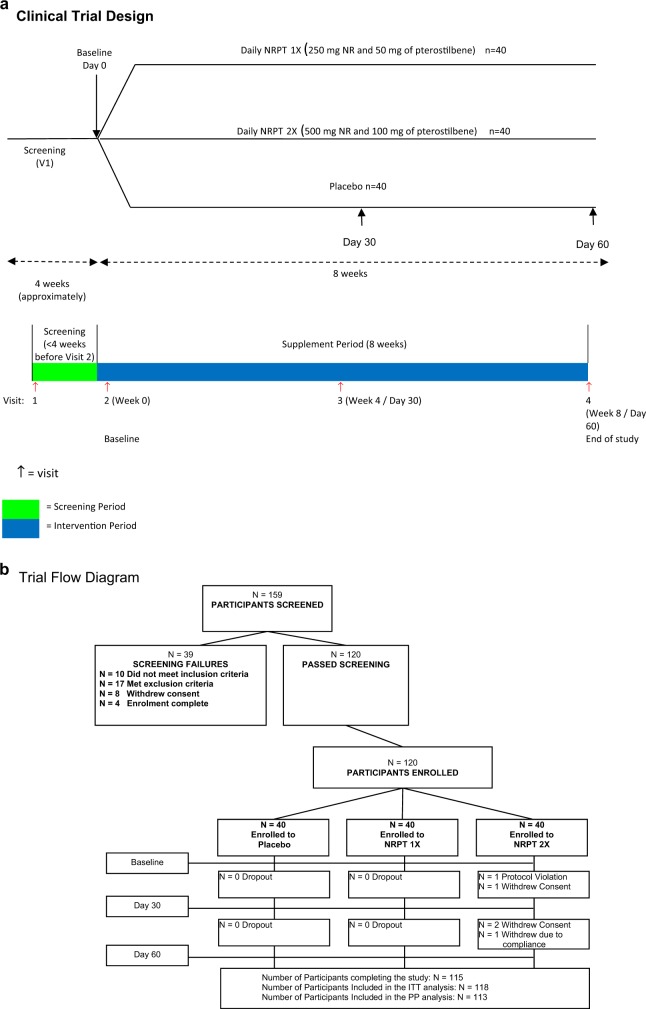
Clinical trial diagrams. **a** Clinical Trial Design diagram. Schematic depicting the randomized, double-blind, placebo controlled, three-arm parallel group study. The study consisted of a single eight-week study period. Clinic visits occurred at day 0 (baseline), day 30, and day 60. Subjects were asked to fast 12 h prior to each clinic visit. Each clinic visit consisted of a physical exam including as well as blood draws to evaluate safety and efficacy endpoints of the trial. **b** Clinical Trial Flow Diagram. Schematic depicting recruitment and disposition of study participants. A total of 159 potential subjects were screened to successfully enroll 120 eligible subjects and randomize them 1:1:1 to the three arms. One-hundred fifteen subjects completed the 60-day study

The safety and efficacy of NRPT, a supplement combining nicotinamide riboside and pterostilbene, was investigated in a population of 120 participants in a randomized, double-blind, placebo-controlled repeat dose clinical trial. This trial consisted of three arms of 40 healthy subjects between the ages of 60 to 80: placebo, NRPT at recommended dose (NRPT 1X; 250 mg of NR plus 50 mg of PT), and NRPT at double dose (NRPT 2X; 500 mg of NR plus 100 mg of PT). Each subject took their assigned treatment orally, at breakfast, daily for 8 weeks. Blood was taken at baseline, at 4 weeks and at 8 weeks to evaluate safety and efficacy in raising NAD^+^ concentrations in whole blood with a 30-day follow-up after supplementation was stopped. A schematic of the study is shown in Fig. 1a.

All participants were analyzed in the Intention-to-Treat Population (ITT), with 40 participants in the NRPT 1X group, 38 in the NRPT 2X group, and 40 in the placebo group. Data collected from the ITT group was analyzed for all safety endpoints. One-hundred and thirteen participants (113) were analyzed in the Per Protocol Population (PP), with 40 participants in the NRPT 1X group, 33 in the NRPT 2X group, and 40 in the placebo group (Fig. 1b). Seven participants were removed from the total population for the Per Protocol analysis; one participant was incorrectly enrolled into the study, three participants withdrew consent, two participants had low investigational product compliance (compliance less than 70%), and one participant was not compliant with study procedures. Data from the PP group was analyzed when a confounding error would clearly be introduced due to lack of protocol compliance. At randomization, participants were well matched between groups and compliance in all groups exceeded 94% (Fig. 1b). The ITT population was used for all analysis except NAD^+^ and mobility analyses, where the PP population was analyzed to eliminate error from non-compliance.

Demographics of participants in the NRPT 1X, NRPT 2X, and placebo group were well matched for age, gender, BMI, smoking status, race, and ethnicity (Table S1). Of 120 randomized participants in the safety population, 87% identified themselves as western European white, between 60 and 79 years of age with a BMI of 18 to 35 kg/m^2^. Sixty-one percent were non-smokers and 68% were female. Use of alcohol was generally evenly distributed between the participants with 91% being either occasional, weekly or daily users but none classified as heavy users (Table S1).

### Adverse events

A total of 66 adverse events (AEs) were reported by 45 participants (Table S2). Of these, 18 AEs were reported by 13 participants in the placebo group, 25 reported by 15 participants in the NRPT 1X group, and 23 reported by 17 participants in the NRPT 2X group. There were no significant differences in the incidence of AEs among groups. There was one AE mild in intensity assessed as possibly related to the placebo product (pruritus), one AE mild in intensity assessed as possibly related to NRPT 1X (nausea), and five AEs (moderate fatigue, mild headache, moderate dyspepsia, moderate abdominal discomfort and diarrhea) reported by five participants in the NRPT 2X group (Table 1). Four of these AEs were assessed as possibly related to NRPT 2X, while one AE (diarrhea) was assessed as probably related to NRPT 2X. All other AEs were classified as unlikely or not related to the investigational product. All participants reporting AEs recovered and there were no serious AEs reported during this clinical study.

**Table 1:** Total number of possibly, probably, or most probably related AEs and number of participants experiencing at least one AE separated by system organ class category

	Placebo (*N* = 40)		NRPT 1X (*N* = 40)		NRPT 2X (*N* = 40)		Between group *p*-value^a^
	Number of AEs	Participants experiencing AEs	Number of AEs	Participants experiencing AEs	Number of AEs	Participants experiencing AEs	
	*n*	*n* (%)	*n*	*n* (%)	*n*	*n* (%)	
Gastrointestinal disorders	0	0 (0%)	1	1 (2.5%)	3	3 (7.5%)	—
General disorders and administration site conditions	0	0 (0%)	0	0 (0%)	1	1 (2.5%)	—
Nervous system disorders	0	0 (0%)	0	0 (0%)	1	1 (2.5%)	—
Skin and subcutaneous tissue disorders	1	1 (2.5%)	0	0 (0%)	0	0 (0%)	—
Overall adverse events	1	1 (2.5%)	1	1 (2.5%)	5	5 (12.5%)	0.088

Probability values* p* ≤ 0.05 are statistically significant

*n* number

^a^ Between group comparisons were made using the Chi-Squared test

### NRPT increases NAD^+^

**Fig. 2:**
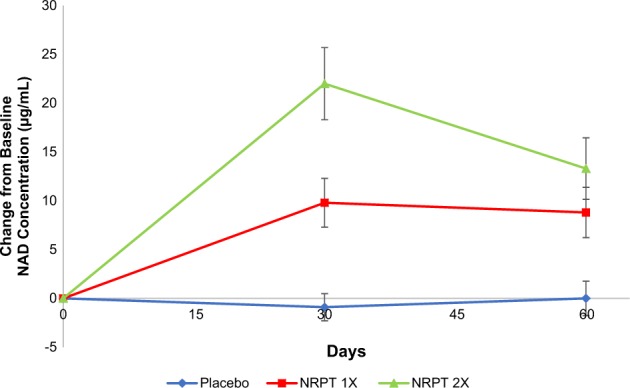
NRPT increases NAD^+^ levels. Total NAD^+^ levels were measured in whole blood from all subjects at day 0 (baseline), day 30, and day 60. Graph depicts change from baseline NAD^+^ concentration (μg/ml) for Placebo (blue diamonds), NRPT 1X (red squares) and NRPT 2X (green triangles). Data shown is the mean ± standard deviation of the mean for each time point

Whole blood was collected at baseline, day 30 and day 60 from all subjects for subsequent NAD^+^ analysis. Collection was at pH 5, which led to red blood cell lysis but preserved NAD^+^ for analysis. A GLP-compliant method was developed to analyze NAD^+^ from human whole-blood lysates by Liquid chromatography–mass spectrometry (LC-MS/MS). As shown in Fig. 2, the placebo group showed no increase of NAD^+^ over the 60-day treatment period. However, NAD^+^ concentrations did significantly increase in a dose-dependent manner at 30 days; NAD^+^ levels increased approximately 40% in the NRPT 1X group and approximately 90% in the NRPT 2X group relative to baseline (Fig. 2; Table S3). The 40% increase in NAD^+^ concentration observed in the NRPT 1X group was sustained at 60 days. The increase in NAD^+^ levels seen in the NRPT 2X group was sustained at approximately 55% over baseline at 60 days. This increase remained significantly higher than the NRPT 1X group at 60 days (Fig. 2; Table S3). The within-group increases in the NRPT 1X and NRPT 2X groups at day 30 and day 60 were significant, as were the differences between groups at those time points (Table S3). Thus, NRPT significantly increases NAD^+^ levels in a sustained way.

### NRPT 1X and liver enzymes

Liver enzymes in blood were also determined as a measure of health of that organ. Liver tests were within normal ranges at baseline for all subjects. There were no changes in the liver function tests for any group (placebo, NRPT 1X, or NRPT 2X) except that a significant decrease was observed in the ALT (alanine transaminase) test at 30 and 60 days within the NRPT 1X as compared to baseline (Table 2). A similar trend that did not reach significance was also observed for AST (aspartate transaminase). Since the presence of liver enzymes in the blood indicates defects in liver health, the data suggest that NRPT 1X may improve liver function in healthy adults. We intend to pursue subsequent human studies to further investigate the role of NRPT on liver health.

**Table 2:** Liver function tests of all participants (*N* = 120)

	Placebo	NRPT 1X	NRPT 2X	Between group
	Mean ±SD (*n*)	Mean ±SD (*n*)	Mean ±SD (*n*)	*p*-value
Bilirubin concentration (μmol/L)				
Day 0 Baseline	11.6 ± 3.7 (40)	11.2 ± 5.8 (40)	11.3 ± 4.4 (40)	0.697^a,b^
Day 30	11.7 ± 4.8 (40)	11.4 ± 6.4 (40)	11.1 ± 3.9 (39)	0.740^a,b^
Day 60 End of Study	11.4 ± 4.1 (40)	10.5 ± 4.6 (40)	11.6 ± 4.2 (37)	0.344^a,b^
Change from Day 0 to Day 30	0.14 ± 2.96 (40)	0.19 ± 2.71 (40)	−0.45 ± 2.79 (39)	0.641^a,c^
Change from Day 0 to Day 60	−0.1 ± 3.0 (40)	−0.7 ± 3.2 (40)	−0.2 ± 3.8 (37)	0.635^a,c^
Aspartate transaminase (U/L)				
Day 0 Baseline	25.5 ± 6.3 (40)	24.4 ± 9.5 (40)	23.9 ± 5.9 (40)	0.328^d^
Day 30	26.1 ± 5.2 (40)	23.8 ± 8.6 (40)	25.1 ± 5.6 (39)	0.004^d^
Day 60 End of Study	26.4 ± 6.1 (40)	23.6 ± 8.7 (40)	25.3 ± 5.7 (37)	0.003^d^
Change from Day 0 to Day 30	0.6 ± 3.9 (40)	−0.6 ± 3.8 (40)	1.1 ± 3.4 (39)	0.047^d^
Change from Day 0 to Day 60	0.8 ± 5.7 (40)	−0.8 ± 4.5 (40)	0.9 ± 3.3 (37)	0.041^d^
Alanine transaminase (U/L)				
Day 0 Baseline	23.4 ± 8.3 (40)	21.6 ± 6.3 (40)	21.9 ± 7.7 (40)	0.584^d^
Day 30	24.4 ± 9.3 (40)	19.9 ± 5.4 (40)	22.4 ± 7.2 (39)	0.033^d^
Day 60 End of study	25.1 ± 11.6 (40)	19.7 ± 5.1 (40)	21.8 ± 7.3 (37)	0.039^d^
Change from Day 0 to Day 30	1.0 ± 5.6 (40)	−1.7 ± 5.2 (40)^e^	0.4 ± 5.1 (39)	0.032^d^
Change from Day 0 to Day 60	1.6 ± 7.8 (40)	−1.9 ± 4.8 (40)^e^	−0.5 ± 5.0 (37)	0.127^d^
Gamma- glutamyltransferase (U/L)				
Day 0 Baseline	22 ± 13 (39)	28 ± 56 (40)	17 ± 11 (40)	0.079^d^
Day 30	24 ± 15 (40)	28 ± 60 (40)	18 ± 10 (39)	0.092^d^
Day 60 End of study	25 ± 18 (40)	27 ± 56 (40)	18 ± 11 (37)	0.125^d^
Change from Day 0 to Day 30	1.8 ± 6.5 (39)	−0.5 ± 9.2 (40)	1.1 ± 4.0 (39)	0.237^d^
Change from Day 0 to Day 60	2.6 ± 6.9 (39)	−1.0 ± 8.8 (40)	0.9 ± 6.4 (37)	0.593^d^

Probability values *p* ≤ 0.05 are statistically significant

^a^ The logarithmic transformation was required to achieve normality

^b^ Between group comparisons were made using ANOVA (no adjustment for baseline)

^c^ Between group comparisons were made using ANCOVA adjusting for baseline

^d^ Between group comparisons were made using the non-parametric Kruskal–Wallis test

^e^ Denotes significant within-group comparisons were made using the paired Student *t*-test

### NRPT 1X and blood pressure

Vital signs, heart rate, and blood pressure were measured in participants. There were no changes relative to baseline at day 30 or 60 in heart rate or blood pressure in any group except in the NRPT 1X group, where diastolic blood pressure decreased significantly at day 60 (Table S4).

### NRPT and mobility

To assess mobility in the study, a 30-second chair stand test and 6-minute walk test were employed. The 30-second chair stand test is used to determine lower body strength in an elderly population where higher numbers are considered beneficial to the individual. The 6-minute walk test measures distance (in meters) an individual can walk at a normal pace for 6 min. Interestingly, the NRPT 2X group showed a significant within-group increase in mobility in both the 30-s chair test (Table S5) and the 6-min walk test (Table S6) at 60 days compared to baseline. There were no differences observed in the placebo or NRPT 1X group.

### NRPT and other blood markers

After 60 days of daily supplementation, there was no significant difference between groups in hematology and clinical chemistry parameters in participants. Specifically, no significant differences between NRPT 1X, NRPT 2X or placebo were observed in hemoglobin, hematocrit, WBC, RBC, mean corpuscular volume, mean corpuscular hemoglobin, counts of platelet, neutrophil, lymphocytes, monocytes, eosinophils, or basophils. Electrolytes (sodium, potassium, and chloride) concentration and kidney function, as measured by creatinine, was similar between groups throughout the study (Table S7).

### NRPT and lipids

Analysis of lipids showed across group differences in triglycerides, but this was entirely due to a decline in the placebo group at day 30 and day 60. Levels of triglycerides within the NRPT 1X and NRPT 2X group showed no significant changes from baseline at day 30 or day 60. The decrease in triglycerides in the placebo group at day 60 compared to baseline resulted in a significant difference between the placebo and the NRPT 1X group at day 60 (Table 3). Total cholesterol and LDL-cholesterol showed within-group increases at day 30 (NRPT 2X) and day 60 (NRPT 1X and NRPT 2X) compared to baseline. The increase in total cholesterol in the NRPT 1X group compared to the placebo group was not significant at day 30 or day 60. The increase in LDL cholesterol in the NRPT 1X group compared to the placebo group was approximately 3% at day 30 and 3.5% at day 60. Larger increases in total cholesterol and LDL cholesterol were observed in the NRPT 2X group. However, there were significant across group differences in total and LDL cholesterol at baseline mainly due to lower levels in the placebo group, confounding the interpretation of the study data.

**Table 3:** Lipid profile of participants at baseline (Day 0), day 30, and day 60 in the ITT population (*N* = 118)

	Placebo	NRPT 1X	NRPT 2X	Between group
	Mean ±SD (*n*)	Mean ±SD (*n*)	Mean ±SD (*n*)	*p*-value
Triglyceride concentration (mmol/L)				
Day 0 Baseline	1.24 ± 0.72 (38)	1.33 ± 0.71 (39)	1.03 ± 0.38 (36)	0.145^a,b^
Day 30	1.15 ± 0.60 (40)	1.42 ± 0.93 (40)	1.03 ± 0.39 (38)	0.038^a,b^
Day 60 End of Study	1.11 ± 0.67 (40)	1.48 ± 0.93 (40)	1.08 ± 0.45 (38)	0.018^a,b^
Change from Day 0 to Day 30	−0.09 ± 0.40 (38)	0.01 ± 0.45 (39)	0.01 ± 0.27 (36)	0.427^a,c^
Change from Day 0 to Day 60	−0.12 ± 0.38 (38)^d^	0.11 ± 0.49 (39)^e^	0.07 ± 0.30 (36)	0.023^a,c^
Total cholesterol concentration (mmol/L)				
Day 0 Baseline	5.09 ± 0.84 (38)	5.58 ± 0.81 (39)^e^	5.21 ± 0.84 (36)	0.029^a,b^
Day 30	5.10 ± 0.89 (40)	5.78 ± 0.87 (40)	5.52 ± 0.84 (38)	0.002^a,b^
Day 60 End of Study	5.15 ± 0.92 (40)	5.86 ± 0.85 (40)	5.70 ± 0.94 (38)	<0.001^a,b^
Change from Day 0 to Day 30	−0.02 ± 0.39 (38)	0.13 ± 0.52 (39)	0.32 ± 0.40 (36)^e^	0.002^a,c^
Change from Day 0 to Day 60	0.05 ± 0.53 (38)	0.22 ± 0.57 (39)	0.51 ± 0.48 (36)^e^	<0.001^a,c^
High-density lipoprotein concentration (mmol/L)				
Day 0 Baseline	1.71 ± 0.54 (38)	1.61 ± 0.44 (39)	1.73 ± 0.51 (36)	0.589^a,b^
Day 30	1.68 ± 0.53 (40)	1.58 ± 0.42 (40)	1.74 ± 0.49 (38)	0.389^a,b^
Day 60 End of Study	1.72 ± 0.56 (40)	1.53 ±0.41 (40)	1.74 ± 0.49 (38)	0.157^a,b^
Change from Day 0 to Day 30	−0.020 ± 0.232 (38)	−0.017 ± 0.153 (39)	−0.003 ± 0.140 (36)	0.879^a,c^
Change from Day 0 to Day 60	0.010 ± 0.209 (38)	−0.070 ± 0.194 (39)	−0.009 ± 0.138 (36)	0.113^a,c^
Low-density lipoprotein concentration (mmol/L)				
Day 0 Baseline	2.81 ± 0.81 (38)	3.37 ± 0.67 (39)^e^	3.01 ± 0.80 (36)	0.004^a,b^
Day 30	2.90 ± 0.81 (40)	3.56 ± 0.73 (40)	3.31 ± 0.78 (38)	<0.001^a,b^
Day 60 End of Study	2.93 ± 0.80 (40)	3.65 ± 0.72 (40)	3.47 ± 0.84 (38)	<0.001^a,b^
Change from Day 0 to Day 30	0.04 ± 0.36 (38)	0.15 ± 0.44 (39)	0.32 ± 0.35 (36)^e^	0.004^a,c^
Change from Day 0 to Day 60	0.10 ± 0.38 (38)	0.24 ± 0.43 (39)^e^	0.48 ± 0.40 (36)^e^	<0.001^a,c^

Probability values *p* ≤ 0.05 are statistically significant

^a^ The logarithmic transformation was required to achieve normality

^b^ Between group comparisons were made using ANOVA (no adjustment for baseline)

^c^ Between group comparisons were made using ANCOVA adjusting for baseline

^d^ Denotes significant within-group comparisons were made using the paired Student t-test

^e^ Denotes significant difference compared to placebo as assessed by the Tukey-Kramer post-hoc test

Thus, we stratified the three treatment groups by BMI and reanalyzed the data (Table 4). Subjects in the NRPT 1X group with normal BMI (18–25) showed no significant increases in LDL cholesterol at day 30 or day 60. Subjects in the NRPT 2X group with normal BMI did show increases in LDL cholesterol at day 30 and day 60. Subjects in the overweight category (BMI 25–32) showed increases in LDL cholesterol at day 30 and day 60 in both the NRPT 1X and NRPT 2X groups. However, overweight subjects in the placebo group also showed a significant increase at day 60. Overall, these findings suggest a small but significant increase in cholesterol may occur at the normal dose of NRPT, at least for people with a higher than normal BMI. Further studies are needed with increased number of subjects to determine if the small changes observed here are real or due to chance.

**Table 4:** Stratifying change in LDL at 30 and 60 days by BMI (normal, overweight, obese) in the ITT Population (*N* = 118)

	Placebo		NRPT 1X		NRPT 2X	
	Mean ±SD (*n*)		Mean ±SD (*n*)		Mean ±SD (*n*)	
	30 Days	60 Days	30 Days	60 Days	30 Days	60 Days
Normal BMI	0.10 ± 0.35 (12)	0.10 ± 0.33 (12)	0.18 ± 0.38 (10)	0.10 ± 0.40 (10)	0.23 ± 0.40**(13)	0.64 ± 0.49**(13)
Overweight BMI	0.04 ± 0.16 (13)	0.16 ± 0.28*(13)	0.27 ± 0.47**(16)	0.46 ± 0.43**(16)	0.33 ± 0.27**(14)	0.50 ± 0.30**(14)
Obese BMI	−0.1 ± 0.34 (13)	0.03 ± 0.48 (13)	−0.03 ± 0.40 (12)	0.13 ± 0.39 (12)	0.24 ± 0.44(8)	0.45 ± 0.43*(8)

*Note*: Red denotes significant difference observed within-group using the paired Student *t*-test

*p ≤ 0.05

**p ≤ 0.01

## Discussion

This study aimed to test NRPT in a rigorous placebo-controlled, double-blind, and randomized 120-person human trial for safety and efficacy. Earlier studies involving a small number of subjects vouched for the safety of the ingredients of NRPT separately, but were not placebo-controlled.^7,25^ This rigorous, larger trial showed that AEs were mild and distributed across the NRPT and placebo groups. Thus, NRPT is deemed to be well tolerated. The major efficacy endpoint of the trial was NAD^+^ concentration, which drives activities of sirtuins and many other processes that promote cellular health and decline with age. Thus, an increase in NAD^+^ stands as a key component of how NRPT is envisaged to improve human health. In a small trial of 12 subjects, a single dose of NR was found to raise NAD^+^ levels in white blood cells over a time course of several hours.^7^ Our trial monitored NAD^+^ levels in whole blood at day 0 (baseline), day 30, and day 60. There was a robust 40% (NRPT 1X) and 90% (NRPT 2X) increase in NAD^+^ at day 30 over baseline, and this was fully sustained at day 60 in the NRPT 1X group and partially declined to 55% over baseline in the NRPT 2X group. The placebo group did not show changes in NAD^+^ levels. It is not clear why the NRPT 2X group showed the partial decline at 60 days, but it is possible that extraordinarily high levels of NAD^+^ can induce homeostatic mechanisms to restrain further increases. One possible mechanism is induction of NAD^+^ degrading enzymes, such as CD38.

A previous report has established that NR is metabolized similarly to nicotinamide in humans with the exception of two additional metabolites generated by NR; namely NAMN and NAAD (both known NAD^+^ precursors).^7^ Furthermore, NR exhibits a similar toxicity profile to nicotinamide in rats at very high doses (e.g., 3000 mg/kg NR).^28^ Since nicotinamide has been studied as a dietary supplement in humans for decades, excellent safety data on long term use is available. The EU Scientific Committee on Food has established an upper limit of 900 mg/day as safe.^28^ Thus, the nicotinamide safety information coupled with our safety data presented here demonstrates that consumption of NR is safe for daily use at reasonable doses.

A series of blood parameters were also measured in all subjects, and most showed no significant changes during the trial. However, three measurements did show differences that reached statistical significance. First, diastolic blood pressure was significantly reduced at day 60 in the NRPT 1X group. This finding is consistent with an earlier small trial showing that pterostilbene alone caused a small decrease in diastolic blood pressure.^29^ Second, the liver enzyme ALT showed a significant decrease in the NRPT 1X group at both day 30 and day 60. A second liver enzyme, AST, showed the same trend at both day 30 and day 60, which did not reach significance. Third, a small increase in total and LDL cholesterol was observed in the NRPT 1X group at day 60 (about 3% over placebo) and larger increases in the NRPT 2X groups. When subjects were stratified by BMI, changes in cholesterol in the NRPT 1X group were absent in the normal BMI subgroup and were confined to the overweight subgroup. One confounding factor in interpreting the cholesterol data is that subjects showed significant differences in total and LDL cholesterol at baseline, due to the vagaries in the randomization of subjects. Furthermore, the normal biological variation for LDL for an individual has been estimated to be 9%.^30^ Thus, it is possible only natural variations in LDL were observed in this trial.

Changes in the liver enzymes and cholesterol biosynthesis draw our attention to possible effects of NRPT on the liver. One possibility is that NRPT 1X improves liver health (e.g., by reducing hepatocyte cell death) and the healthier liver shows improved functions, including modestly more synthesis of cholesterol. It is unclear if the small increase in LDL cholesterol is due to an increase in particle size, which would be benign with regards to cardiovascular health. As an example, omega-3-fatty acids raise LDL cholesterol via increases in particle size, but may be beneficial for cardiovascular health.^31,32^ Determination of apoB and apoC3 levels may shed light on this question. Another possibility for the small increases in cholesterol relates to the known function of SIRT1 in promoting reverse cholesterol transport from cells. If NRPT 1X upregulates SIRT1 as expected, an increase in reverse cholesterol transport (e.g., export from cells) could account for the slightly higher levels of cholesterol in blood. In this case, the prognosis would be positive, since lower cholesterol in macrophages should slow their progression to foam cells, which are the initiators of atherosclerotic plaques. Future trials will be necessary to determine whether the cholesterol (and ALT) effects are reproducible.

With respect to mobility, the finding that NRPT 2X group demonstrated significant increase in both the 30-second chair stand and the 6-min walk test at 60 days suggests that prolonged supplementation with NRPT may support overall muscle health and/or energy in an older population. While encouraging, further study is warranted to elucidate mechanisms behind this observed increase in mobility. These studies would include a larger study population observed for a longer period of time to reduce the possibility of seeing correlations by chance.

A tangible strength of this study is in the demonstration that NAD^+^ levels in whole blood can be significantly increased in humans in a safe and sustainable way by oral supplementation with NRPT. Limitations of this study include that only an older population was examined and that potentially promising results from secondary and exploratory endpoints (with the exception of NAD^+^ levels) were not sufficiently powered to make unequivocal conclusions. Still, this study represents an important first step that future clinical studies can build upon.

In summary, we have conducted the first placebo-controlled, randomized and double-blind human trial on NRPT and found that it is well tolerated and significantly raises NAD^+^ levels in circulating blood in a sustained way. Other parameters measured in the trial provide a foundation for future trials, for example, by suggesting that NRPT may have salutary effects on liver function and mobility. We note that there is ample preclinical data in mice and rats that SIRT1 activation, including activation by polyphenols and by NAD^+^ precursors, has beneficial effects on liver function,^5,33,34^ providing further impetus to conduct follow-up trials. The preclinical data also shows beneficial effects of NAD^+^ precursors and polyphenols on muscle disorders, diabetes, and cardiovascular, and brain health. Whether some of these benefits will be observed in humans is a question now open to investigation.

## Methods

### Clinical trial

This human clinical trial was conducted in accordance with the ethical principles that have their origins in the Declaration of Helsinki and its subsequent amendments (clinical trials.gov identifier NCT02678611) This study was reviewed by the Natural and Non-prescription Health Products Directorate (NNHPD), Health Canada and a research ethics board. Notice of authorization was granted on 11 December 2015 by the NNHPD, Ottawa, Ontario and unconditional approval was granted on 23 December 2015 by the Institutional Review Board (IRB Services, Aurora, Ontario)

This study was a randomized, double-blinded, placebo-controlled study carried out at three sites. The testing sites were in London, Ontario (Canada), Orlando, Florida, and Irvine, California. The intervention phase was 8 weeks with a 30-day follow-up period. All participants that met inclusion and not exclusion criteria at screening were randomized into three arms: placebo, recommended dose of NRPT (NRPT 1X), and double the recommended dose of NRPT (NRPT 2X). Study recruited subjects beginning in January 2016 and had completed the in-human phase of the trial by July 2016 after full enrollment and enough time for all subject to complete protocol.

The primary objective of this study was to evaluate the safety and tolerability of two doses of NRPT in elderly participants during and after eight weeks of treatment. Safety parameters measured included a standard clinical checkup, self-reported AEs, complete blood count (CBC), electrolytes (Na, K, Cl), kidney function (creatinine), and liver function (AST, ALT, GGT and bilirubin). Secondary objectives of the study included evaluations of potential benefits of NRPT in increasing the concentration of NAD^+^ in the blood and improving lipid metabolism.

### Participants

The inclusion criteria were as follows: males and females between the ages of 60 and 80 with a body mass index (BMI) between 18 to 35 kg/m^2^ (±1 kg/m^2^). Participants agreed to avoid taking vitamin B_3_ (nicotinic acid, nicotinamide, or nicotinamide riboside) supplements or multivitamins 14 days prior to randomization and for the duration of the study period. Participants were healthy as determined by laboratory results, medical history, and physical examination. Individuals gave voluntary, written, and informed consent to participate in the study.

Individuals were excluded if they met any of these characteristics: unstable medical conditions, history of any significant chronic disease or any clinically active illness within 3 months of study entry, history of renal or liver impairment, any endocrine, inflammatory, cardiovascular, gastrointestinal, neurological, psychiatric, neoplastic or metabolic disease, significant or untreated medical disorders including recent myocardial ischemia or infarction, unstable angina, uncontrolled hypertension, AIDS, malignancy, epilepsy, and recent cerebrovascular disease, recently experienced a traumatic injury, infections or undergone surgery, history of pellagra or niacin deficiency, currently taking lipid lowering drugs, use of natural health products containing nicotinamide riboside within 14 days prior to randomization and during the study. Participants with a history of, or current diagnosis of any cancer (except for successfully treated basal cell carcinoma) diagnosed less than five years prior to screening were also excluded. Volunteers with cancer in full remission more than five years after diagnosis were accepted. Subjects were also excluded if they had participated in any clinical trial with an investigational medicinal product within the past three months prior to the first dose in the current study, alcohol use of more than two standard alcoholic drinks per day, history of alcoholism or drug abuse within one year prior to screening, history of significant allergies, allergy or sensitivity to any of the investigational product ingredients, or used medicinal marijuana. Subjects were excluded if they reported clinically significant abnormal laboratory results at screening or were cognitively impaired and/or who are unable to give informed consent were also excluded.

### Intervention

The investigational product NRPT contained 125 mg of NR and 25 mg of pterostilbene per capsule. Each capsule also contained the non-dietary ingredients microcrystalline cellulose, silicon dioxide, magnesium stearate, gelatin. Placebo capsules consisted of microcrystalline cellulose, silicon dioxide, magnesium stearate, gelatin. During the intervention period, two groups received the investigational supplement while the third group received placebo capsules. All subjects took four capsules daily. All participants received two bottles containing capsules (Bottle A and Bottle B) and were instructed to take two capsules from each bottle daily. NRPT 1X arm was provided with Bottle A containing NRPT and Bottle B containing placebo capsules; NRPT 2X arm was provided with Bottle A containing NRPT and Bottle B containing NRPT capsules. The matched placebo pills and the investigational product (NRPT) were provided by Elysium Health (New York, NY).

### Randomization and blinding

A randomization schedule was prepared using block randomization by an unblinded person at the study site who was not involved in study assessment. random allocation sequence was generated using www.randomization.com. A random sequence of treatments was generated using 20 blocks of 6 (use seed #12081 to pull on website). This random sequence of treatments was then associated with a random permutation of 120 numbers from 616,001 to 616,120 (use seed #7123). The investigational product, NRPT, and placebo were sealed in identical bottles, which were labeled per the requirements of ICH-GCP guidelines and applicable local regulatory guidelines. The placebo capsules mimicked the size, shape, and color of the investigational product capsules. The investigational product was labeled by unblinded personnel at KGK Synergize who were not involved in study assessments. All clinic staff involved in product dispensing, collection of data, and monitoring charts and analysis of outcomes remained blinded for the duration of the study.

### Clinic visits

Eligible volunteers returned to the clinic in the morning, after a 12-hour fast (no food or drink except water) for baseline assessments. A physical exam was conducted where weight was measured and BMI calculated. Resting blood pressure and heart rate measurements were also taken. Fasting blood samples were collected for fasting glucose, lipid panel, hs-CRP, CBC, electrolytes (Na, K, Cl), creatinine, AST, ALT, GGT, bilirubin, PBMC, and NAD^+^ analysis.

### Sample collection and preparation for NAD^+^ analysis

Prior to blood collection, 1 mL of 0.5 M perchloric acid (PCA) was aliquoted to four cryogenic screw cap bottles with seals and placed on wet ice. Fasting 4 mL whole-blood samples were collected in sodium citrate tubes for analysis of nicotinamide adenine dinucleotide (NAD^+^). The tubes were inverted gently four times and the placed immediately on wet ice. Whole-blood aliquots of 0.1 mL of were then transferred to each cryovial and gently inverted four times and then placed on wet ice. This treatment lysed all the blood cells. Lastly, the screw caps were replaced and the tubes kept on ice were then stored at −80 °C until analyzed.

### NAD^+^ analysis from whole-blood lysate

Samples were thawed and centrifuged at 13,000 rpm for 5 min at room temperature. In all, 0.11 mL of supernatant was transferred to 2.0 mL glass HPLC injection vial. Then 100 µL of 0.5 M PCA in water was added. Fifty microliters of internal standard solution (5 µg/mL of ^13^C5-nicotinamide adenine dinucleotide in 0.5 M PCA) was then added followed by 0.5 mL of 0.5 M PCA in water. Samples were capped and vortexed for 20 s. Ten microliters was then injected onto the LC/MS/MS to quantitate NAD^+^ using an isotopically labeled d5-NAD^+^ as an internal standard. Mobile phase A was 0.5% formic acid in water and mobile phase B was 0.5% formic acid in acetonitrile. A linear gradient of 0–100%B was run and the mass spec was set on positive ion mode looking for the transitions of 664.4 → 524.0 (NAD^+^) and 669.4 → 529.3 (the internal standard).

### Statistics

Numerical efficacy endpoints were formally tested for significance between groups by analysis of covariance (ANCOVA). The dependent variable was the value at end-of-study (day 60); the factor was the product group, and the value at baseline (day 0) was the covariate. When the omnibus ANCOVA and ANOVA *p*-values suggested at least one mean difference was present, pairwise comparisons using the Tukey-Kramer procedure were run. Significant efficacy of NRPT, relative to placebo, was inferred if the pairwise comparisons were significantly different from zero (*p* ≤ 0.05). Intractably non-normal data was formally tested for significance between groups by the Kruskal–Wallis test. When the omnibus Kruskal–Wallis *p*-values suggested at least one mean difference was present, pairwise comparisons using the Bonferroni adjusted Mann–Whitney tests were run. Significant efficacy of NRPT, relative to placebo, was inferred if the pairwise comparisons were significantly different from zero (*p* ≤ 0.05). A within-group analysis on efficacy endpoints was done using a Student’s paired samples *t*-test or, in instances of intractable non-normality, Wilcoxon sign rank test. All missing values in the intent-to-treat (effectiveness) analysis was imputed with the most recent previously-available value (LOCF, or “last-observation-carried-forward” imputation). No imputation will be performed for missing values of safety variables. No changes in methods of analysis were made after unblinding occurred. However, sensitivity analysis was performed on all datasets that included LOCF to ensure that imputation of missing values did not significantly impact outcomes. Our sensitivity analysis did not find a case where imputation altered the significance of an association (analysis not shown).

### Data availability statement

The datasets generated and/or analyzed during this study are available from the corresponding author on reasonable request.

## Electronic supplementary material

supplementary table 1

Supplementary Table 2

Supplementary Table 3

Supplementary Table 4

Supplementary Table 5

Supplementary Table 6

Supplementary Table 7

## Electronic supplementary material

**Supplementary information** accompanies the paper on the *npj Aging and Mechanisms of Disease* website (10.1038/s41514-017-0016-9).
