# Author Correction: Repeat dose NRPT (nicotinamide riboside and pterostilbene) increases NAD^+^ levels in humans safely and sustainably: a randomized, double-blind, placebo-controlled study

**DOI:** 10.1038/s41514-018-0027-1

**Published:** 2018-08-20

**Authors:** Ryan W. Dellinger, Santiago Roel Santos, Mark Morris, Mal Evans, Dan Alminana, Leonard Guarente, Eric Marcotulli

**Affiliations:** 1 Elysium Health, Inc, New York, NY USA; 2 KGK Synergize London, London, ON Canada; 3 Department of Biology, MIT, 77 Massachusetts avenue, 68-280, Cambridge, MA 02139 USA

**Correction to:** *npj Aging and Mechanisms of Disease* **3**, 17 (2017); 10.1038/s41514-017-0016-9; Published online 24 November 2017

The original version of the published article contained an incorrect citation for reference 20 “Hubbard, B. P. & Sinclair, D. A. Measurement of sirtuin enzyme activity using a substrate-agnostic fluorometric nicotinamide assay. *Methods Mol. Biol*. **1077**, 167–177 (2013).” The citation for reference 20 has been changed to “Cheng, Y. et al. SIRT1 activation by pterostilbene attenuates the skeletal muscle oxidative stress injury and mitochondrial dysfunction induced by ischemia reperfusion injury. *Apoptosis* **21**, 905–916 (2016).” The original version of the published article did not list a source for the placebo pills or the investigational product NRPT in the Intervention section of Methods. The last sentence of the Intervention section of Methods now lists the source for the placebo pills and the investigational product NRPT as follows: “The matched placebo pills and the investigational product (NRPT) were provided by Elysium Health (New York, NY).” This has now been corrected in the PDF and HTML versions of the article.

