# Supplementary material for: Repeat dose NRPT (nicotinamide riboside and pterostilbene) increases NAD+ levels in humans safely and sustainably: a randomized, double-blind, placebo-controlled study
Source: NPJ Aging Mech Dis. 2017 Nov 24;3:17. doi: 10.1038/s41514-017-0016-9 (PMC5701244; doi:10.1038/s41514-017-0016-9)
Supplement: Supplementary file 1 — supplementary table 1 [file 41514_2017_16_MOESM1_ESM.docx]

Table S1: Demographics for Participants Enrolled in the Study (N = 120).

|  | **All Participants**  **(N = 120)** | **Placebo**  **(N = 40)** | **NRPT 1X**  **(N = 40)** | **NRPT 2X**  **(N = 40)** | **P Value** ^σ^ |
| --- | --- | --- | --- | --- | --- |
| **Age (Years)**  Mean ± SD | 66.8 ± 5.2 | 66.4 ± 5.3 | 67.5 ± 5.1 | 66.3 ± 5.3 | 0.494 § |
| **BMI (kg/m^2^)**  Mean ± SD | 27.6 ± 4.1 | 28.0 ± 4.1 | 27.9 ± 4.1 | 26.9 ± 4.0 | 0.424 § |
| **Gender [n (%)]** Female Male | 82 (68%) 38 (32%) | 24 (60%) 16 (40%) | 30 (75%) 10 (25%) | 28 (70%) 12 (30%) | 0.389 |
| **Alcohol Use [n (%)]** None Occasionally Weekly  Daily | 11 (9%) 23 (19%) 48 (40%)  38 (32%) | 7 (18%) 7 (18%) 15 (38%)  11 (28%) | 2 (5%) 8 (20%) 14 (35%)  16 (40%) | 2 (5%) 8 (20%) 19 (48%)  11 (28%) | 0.417 |
| **Smoking Status [n (%)]** Current Smoker Ex-Smoker Non-Smoker | 8 (7%) 39 (32%) 73 (61%) | 3 (8%)  13 (32%) 24 (60%) | 3 (8%) 13 (32%) 24 (60%) | 2 (5%) 13 (32%) 25 (62%) | 1.000 |
| **Race [n (%)]** Black or African American Eastern European White  Hispanic or Latino Other Western European White | 2 (2%) 9 (8%) 3 (2%) 2 (1%) 104 (87%) | 2 (5%) 4 (10%) 1 (2%) 1 (2%) 32 (80%) | 0 (0%) 3 (8%) 1 (2%) 1 (0%) 35 (88%) | 0 (0%) 2 (5%) 1 (2%) 0 (0%) 37 (92%) | 0.662 |
| Max, maximum; Min, minimum; N, number; %, percentage; SD, standard deviation.  σ Between group comparisons were made using the Chi-Squared test  Probability values P≤0.05 are statistically significant. | | | | | |
