# Supplementary material for: Repeat dose NRPT (nicotinamide riboside and pterostilbene) increases NAD+ levels in humans safely and sustainably: a randomized, double-blind, placebo-controlled study
Source: NPJ Aging Mech Dis. 2017 Nov 24;3:17. doi: 10.1038/s41514-017-0016-9 (PMC5701244; doi:10.1038/s41514-017-0016-9)
Supplement: Supplementary file 2 — Supplementary Table 2 [file 41514_2017_16_MOESM2_ESM.docx]

Table S2: Total Number of AEs and Number of Participants Experiencing at Least One AE Separated by System Organ Class Category

|  | **Placebo**  **(N = 40)** | | **NRPT 1X**  **(N = 40)** | | **NRPT 2X**  **(N = 40)** | | **Between Group**  **P-Value ^σ^** |
| --- | --- | --- | --- | --- | --- | --- | --- |
|  | **Number of AEs** | **Participants**  **Experiencing AEs** | **Number of AEs** | **Participants**  **Experiencing AEs** | **Number of AEs** | **Participants**  **Experiencing AEs** |  |
|  | **n** | **n (%)** | **n** | **n (%)** | **n** | **n (%)** |  |
| **Cardiac disorders** | 0 | 0 (0%) | 1 | 1 (2.5%) | 0 | 0 (0%) | - |
| **Gastrointestinal disorders** | 3 | 2 (5.0%) | 4 | 3 (7.5%) | 8 | 8 (20.0%) | - |
| **General disorders and**  **administration site conditions** | 2 | 2 (5.0%) | 2 | 2 (5.0%) | 1 | 1 (2.5%) | - |
| **Immune system disorders** | 0 | 0 (0%) | 1 | 1 (2.5%) | 0 | 0 (0%) | - |
| **Infections and infestations** | 3 | 3 (7.5%) | 8 | 7 (17.5%) | 4 | 4 (10.0%) | - |
| **Investigations** | 1 | 1 (2.5%) | 0 | 0 (0%) | 0 | 0 (0%) | - |
| **Metabolism and nutrition disorders** | 1 | 1 (2.5%) | 0 | 0 (0%) | 0 | 0 (0%) | - |
| **Musculoskeletal and connective tissue disorders** | 3 | 3 (7.5%) | 2 | 1 (2.5%) | 1 | 1 (2.5%) | - |
| **Nervous system disorders** | 2 | 2 (5.0%) | 3 | 2 (5.0%) | 2 | 2 (5.0%) | - |
| **Psychiatric disorders** | 0 | 0 (0%) | 1 | 1 (2.5%) | 2 | 2 (5.0%) | - |
| **Renal and urinary disorders** | 0 | 0 (0%) | 1 | 1 (2.5%) | 1 | 1 (2.5%) | - |
| **Respiratory, thoracic and mediastinal disorders** | 2 | 2 (5.0%) | 0 | 0 (0%) | 2 | 2 (5.0%) | - |
| **Skin and subcutaneous tissue disorders** | 1 | 1 (2.5%) | 0 | 0 (0%) | 2 | 2 (5.0%) | - |
| **Surgical and medical procedures** | 0 | 0 (0%) | 1 | 1 (2.5%) | 0 | 0 (0%) | - |
| **Vascular disorders** | 0 | 0 (0%) | 1 | 1 (2.5%) | 0 | 0 (0%) | - |
| **Overall Adverse Events** | 18 | 13 (32.5%) | 25 | 15 (37.5%) | 23 | 17 (45.0%) | 0.512 |
| n, number.  σ Between group comparisons were made using the Chi-Squared test | | | | | | | |
