# Supplementary material for: Repeat dose NRPT (nicotinamide riboside and pterostilbene) increases NAD+ levels in humans safely and sustainably: a randomized, double-blind, placebo-controlled study
Source: NPJ Aging Mech Dis. 2017 Nov 24;3:17. doi: 10.1038/s41514-017-0016-9 (PMC5701244; doi:10.1038/s41514-017-0016-9)
Supplement: Supplementary file 3 — Supplementary Table 3 [file 41514_2017_16_MOESM3_ESM.docx]

**Table S3: Basis increases NAD^+^ in the PP Population (N = 113).**

|  | **Placebo** | **NRPT 1X** | **NRPT 2X** | **Between Group**  **P-Value** |
| --- | --- | --- | --- | --- |
|  | **Mean ±SD (n)** | **Mean ±SD (n)** | **Mean ±SD (n)** |  |
| **NAD Concentration (μg/mL)** | | | | |
| **Day 0**  **Baseline** | 22.0 ± 8.1 (40) | 22.5 ± 11.9 (39) | 23.8 ± 9.0 (38) | 0.619^λ^ § |
| **Day 30** | 21.1 ± 8.6 (40) | 32.2 ± 13.4 (40) | 45.8 ± 20.8 (38) | <**0.001**^λ^ § |
| **Day 60**  **End of Study** | 22.0 ± 7.8 (40) | 31.5 ± 16.3 (40) | 37.2 ± 16.7 (38) | <**0.001**^λ^ § |
| **Change from**  **Day 0 to Day 30** | -0.9 ± 8.8 (40) | 9.8 ± 15.6 (39) ^a^ | 22.0 ± 22.8 (38) ^a^ | <**0.001**^λ^ Δ |
| **Change from**  **Day 0 to Day 60** | 0.0 ± 11.2 (40) | 8.8 ± 16.1 (39) ^a^ | 13.3 ± 19.4 (38) ^a^ | <**0.001**^λ^ Δ |

§ Between group comparison were made using ANOVA.

Δ Between group comparisons were made using ANCOVA adjusting for baseline.

^λ^The square root transformation was required to achieve normality for all change from Day 0 to Day 30 and Change from Day 0 to Day 60 data.

^a^ denotes significant difference compared to placebo as assessed by the Tukey-Kramer post-hoc test.

Probability values P≤0.05 are statistically significant.
