# Supplementary material for: Repeat dose NRPT (nicotinamide riboside and pterostilbene) increases NAD+ levels in humans safely and sustainably: a randomized, double-blind, placebo-controlled study
Source: NPJ Aging Mech Dis. 2017 Nov 24;3:17. doi: 10.1038/s41514-017-0016-9 (PMC5701244; doi:10.1038/s41514-017-0016-9)
Supplement: Supplementary file 4 — Supplementary Table 4 [file 41514_2017_16_MOESM4_ESM.docx]

Table S4: Vital Signs of All Participants Randomized in the Study at Day 0, Day 30 and Day 60 (N = 120).

|  | **Placebo** | **NRPT 1X** | **NRPT 2X** | **Between Group**  **P Value** Δ |
| --- | --- | --- | --- | --- |
|  | **Mean ±SD (n)** | **Mean ±SD (n)** | **Mean ±SD (n)** |  |
| **Systolic Blood Pressure (mmHg)** | | | | |
| **Day 0**  **Baseline** | 125.9 ± 14.2 (40) | 125.6 ± 15.9 (40) | 124.3 ± 16.0 (40) | 0.884 § |
| **Day 30** | 123.9 ± 18.3 (40) | 125.9 ± 20.3 (40) | 124.3 ± 17.3 (38) | 0.877 § |
| **Day 60**  **End of Study** | 125.8 ± 13.4 (40) | 122.4 ± 19.2 (40) | 123.1 ± 15.6 (38) | 0.617 § |
| **Change from**  **Day 0 to Day 30** | -2.0 ± 13.5 (40) | 0.3 ± 12.8 (40) | -0.1 ± 12.2 (38) | 0.711 Δ |
| **Change from**  **Day 0 to Day 60** | -0.1 ± 10.7 (40) | -3.2 ± 14.0 (40) | -0.9 ± 11.1 (38) | 0.462 Δ |
| **Diastolic Blood Pressure (mmHg)** | | | | |
| **Day 0**  **Baseline** | 76.4 ± 10.5 (40) | 76.3 ± 9.8 (40) | 75.2 ± 8.0 (40) | 0.815 § |
| **Day 30** | 75.6 ± 10.8 (40) | 75.5 ± 12.2 (40) | 75.5 ± 9.1 (38) | 0.998 § |
| **Day 60**  **End of Study** | 76.1 ± 10.1 (40) | 72.9 ± 10.1 (40) | 75.0 ± 9.2 (38) | 0.346 § |
| **Change from**  **Day 0 to Day 30** | -0.8 ± 7.4 (40) | -0.7 ± 9.1 (40) | 0.6 ± 7.4 (38) | 0.804 Δ |
| **Change from**  **Day 0 to Day 60** | -0.3 ± 8.2 (40) | -3.4 ± 7.1 (40)^b^ | -0.1 ± 7.0 (38) | 0.092 Δ |
| **Heart Rate (BPM)** | | | | |
| **Day 0**  **Baseline** | 65.6 ± 8.0 (40) | 70.3 ± 10.9 (40) | 67.3 ± 10.5 (40) | 0.104 § |
| **Day 30** | 66.7 ± 9.2 (40) | 68.5 ± 10.2 (40) | 66.1 ± 9.2 (38) | 0.532 § |
| **Day 60**  **End of Study** | 67.6 ± 10.0 (40) | 68.7 ± 9.6 (40) | 64.9 ± 8.3 (38) | 0.186 § |
| **Change from**  **Day 0 to Day 30** | 1.1 ± 6.8 (40) | -1.9 ± 8.1 (40) | -0.0 ± 8.9 (38) | 0.708 Δ |
| **Change from**  **Day 0 to Day 60** | 2.0 ± 7.6 (40) | -1.7 ± 8.0 (40) | -1.5 ± 8.1 (38) | 0.146 Δ |
| BPM, beats per minute; kg, kilograms; Max, maximum; mmHg, m, meter;  mmHg, millimeters of mercury; Min, minimum; N, number; SD, standard deviation.  § Between group comparisons were made using ANOVA.  Δ Between group comparisons were made using ANCOVA adjusting for baseline.  ^b^ Denotes significant within group comparisons were made using the paired Student t-test  Probability values P≤0.05 are statistically significant. | | | | |
