# Supplementary material for: Repeat dose NRPT (nicotinamide riboside and pterostilbene) increases NAD+ levels in humans safely and sustainably: a randomized, double-blind, placebo-controlled study
Source: NPJ Aging Mech Dis. 2017 Nov 24;3:17. doi: 10.1038/s41514-017-0016-9 (PMC5701244; doi:10.1038/s41514-017-0016-9)
Supplement: Supplementary file 5 — Supplementary Table 5 [file 41514_2017_16_MOESM5_ESM.docx]

Table S5: Physical Performance Measured by the 30 Second Chair Stand Test at Baseline (Day 0), Day 30, and Day 60 for Participants in the PP Population (N = 113).

|  | **Placebo** | **NRPT 1X** | **NRPT 2X** | **Between Group**  **P-Value** |
| --- | --- | --- | --- | --- |
|  | **Mean ±SD (n)** | **Mean ±SD (n)** | **Mean ±SD (n)** |  |
| **Number of Stands from Chair in 30 Seconds** | | | | |
| **Day 0**  **Baseline** | 13.2 ± 4.5 (40) | 13.0 ± 3.1 (40) | 13.4 ± 3.5 (33) | 0.896* § |
| **Day 30** | 13.5 ± 4.4 (40) | 12.9 ± 3.2 (40) | 14.0 ± 3.8 (33) | 0.612* § |
| **Day 60**  **End of Study** | 13.6 ± 5.4 (40) | 13.2 ± 3.4 (40) | 14.4 ± 4.2 (33) | 0.470* § |
| **Change from**  **Day 0 to**  **Day 30** | 0.32 ± 2.03 (40) | -0.05 ± 2.28 (40) | 0.64 ± 1.54 (33) | 0.450*^∆^ |
| **Change from**  **Day 0 to**  **Day 60** | 0.50 ± 3.08 (40) | 0.28 ± 2.72 (40) | 1.03 ± 2.14 (33)^b^ | 0.406*^∆^ |
| n, number; SD, standard deviation; Min, minimum; Max, maximum.  § Between group comparisons were made using ANOVA (no adjustment for baseline)  Δ Between group comparisons were made using ANCOVA adjusting for baseline.  δ Within group comparisons were made using the paired Student t-test.  ^b^ Denotes significant within group comparisons were made using the paired Student t-test  * The logarithmic transformation was required to achieve normality  Probability values P≤0.05 are statistically significant. | | | | |
