# Supplementary material for: Repeat dose NRPT (nicotinamide riboside and pterostilbene) increases NAD+ levels in humans safely and sustainably: a randomized, double-blind, placebo-controlled study
Source: NPJ Aging Mech Dis. 2017 Nov 24;3:17. doi: 10.1038/s41514-017-0016-9 (PMC5701244; doi:10.1038/s41514-017-0016-9)
Supplement: Supplementary file 6 — Supplementary Table 6 [file 41514_2017_16_MOESM6_ESM.docx]

Table S6: Physical Performance Measured by the 6 Minute Walk Test at Baseline (Day 0), Day 30, and Day 60 for Participants in the PP Population (N = 113).

|  | **Placebo** | **NRPT 1X** | **NRPT 2X** | **Between Group**  **P-Value** |
| --- | --- | --- | --- | --- |
|  | **Mean ±SD (n)** | **Mean ±SD (n)** | **Mean ±SD (n)** |  |
| **Distance Walked (m)** | | | | |
| **Day 0**  **Baseline** | 459 ± 79 (40) | 436 ± 96 (40) | 455 ± 69 (33) | 0.422 § |
| **Day 30** | 466 ± 87 (40) | 425 ± 103 (40) | 470 ± 73 (33) | 0.053 § |
| **Day 60**  **End of Study** | 475 ± 78 (40) | 429 ± 89 (40) | 489 ± 74 (33) | 0.005 § |
| **Change from**  **Day 0 to Day 30** | 7 ± 64 (40) | -11 ± 90 (40) | 16 ± 46 (33) | 0.105^∆^ |
| **Change from**  **Day 0 to Day 60** | 16 ± 58 (40) | -7 ± 98 (40) | 34 ± 64 (33)^b^ | **0.007^∆^** |
| n, number; Min, minimum; Max, maximum; SD, standard deviation.  § Between group comparisons were made using ANOVA (no adjustment for baseline)  Δ Between group comparisons were made using ANCOVA adjusting for baseline.  δ Within group comparisons were made using the paired Student t-test.  ^b^ Denotes significant within group comparisons were made using the paired Student t-test  Probability values P≤0.05 are statistically significant. | | | | |
