# Supplementary material for: Repeat dose NRPT (nicotinamide riboside and pterostilbene) increases NAD+ levels in humans safely and sustainably: a randomized, double-blind, placebo-controlled study
Source: NPJ Aging Mech Dis. 2017 Nov 24;3:17. doi: 10.1038/s41514-017-0016-9 (PMC5701244; doi:10.1038/s41514-017-0016-9)
Supplement: Supplementary file 7 — Supplementary Table 7 [file 41514_2017_16_MOESM7_ESM.docx]

Table S7: Haematology and Clinical Chemistry Parameters of All Randomized Participants at Baseline (Day 0), and Day 30, and Day 60 (N = 120).

|  | **Placebo** | **NRPT 1X** | **NRPT 2X** | **Between Group**  **P Value** |
| --- | --- | --- | --- | --- |
|  | **Mean ±SD (n)** | **Mean ±SD (n)** | **Mean ±SD (n)** |  |
| **Hemoglobin Concentration (g/L)** | | | | |
| **Day 0**  **Baseline** | 140.4 ± 14.4 (40) | 136.8 ± 11.7 (40) | 140.4 ± 13.5 (40) | 0.382 § |
| **Day 30** | 139.6 ± 12.6 (39) | 136.5 ± 11.2 (40)) | 139.6 ± 11.4 (38) | 0.401 § |
| **Day 60**  **End of Study** | 138.8 ± 13.1 (40) | 135.5 ± 11.7 (40) | 139.8 ± 11.2 (37) | 0.268 § |
| **Change from**  **Day 0 to Day 30** | -1.2 ± 6.0 (39) | -0.3 ± 4.8 (40) | -0.2 ± 4.2 (38) | 0.685 Δ |
| **Change from**  **Day 0 to Day 60** | -1.6 ± 6.0 (40) | -1.4 ± 5.1 (40) | -0.4 ± 4.9 (37) | 0.417 Δ |
| **Hematocrit (L/L)** | | | | |
| **Day 0**  **Baseline** | 0.414 ± 0.040 (40) | 0.406 ± 0.032 (40) | 0.417 ± 0.037 (40) | 0.392 § |
| **Day 30** | 0.413 ± 0.035 (39) | 0.405 ± 0.029 (40) | 0.414 ± 0.029 (38) | 0.372 § |
| **Day 60**  **End of Study** | 0.410 ± 0.037 (40) | 0.405 ± 0.029 (40) | 0.412 ± 0.029 (37) | 0.593 § |
| **Change from**  **Day 0 to Day 30** | -0.0019 ± 0.0168 (39) | -0.0008 ± 0.0141 (40) | -0.0004 ± 0.0116 (38) | 0.780 Δ |
| **Change from**  **Day 0 to Day 60** | -0.0039 ± 0.0165 (40) | -0.0013 ± 0.0150 (40) | -0.0038 ± 0.0119 (37) | 0.917 Δ |
| **White Blood Cell Count (x E9/L)** | | | | |
| **Day 0**  **Baseline** | 5.70 ± 1.68 (40) | 6.02 ± 1.81 (40) | 5.88 ± 2.26 (40) | 0.794* § |
| **Day 30** | 5.48 ± 1.55 (39) | 5.91 ± 1.54 (40) | 5.30 ± 1.31 (38) | 0.241* § |
| **Day 60**  **End of Study** | 5.63 ± 1.83 (40) | 5.76 ± 1.41 (40) | 5.33 ± 1.48 (37) | 0.442* § |
| **Change from**  **Day 0 to Day 30** | -0.23 ± 0.90 (39) | -0.11 ± 1.65 (40) | -0.57 ± 1.93 (38) | 0.176* Δ |
| **Change from**  **Day 0 to Day 60** | -0.06 ± 0.96 (40) | -0.26 ± 1.15 (40) | -0.51 ± 2.02 (37) | 0.319* Δ |

S4 Table Continued: Haematology and Clinical Chemistry Parameters of All Randomized Participants at Baseline (Day 0), and Day 30, and Day 60 (N = 120).

|  | **Placebo** | **Basis 1X** | **Basis 2X** | **Between Group**  **P Value** |
| --- | --- | --- | --- | --- |
|  | **Mean ±SD (n)** | **Mean ±SD (n)** | **Mean ±SD (n)** |  |
| **Red Blood Cell Count (x E12/L)** | | | | |
| **Day 0**  **Baseline** | 4.58 ± 0.46 (40) | 4.56 ± 0.36 (40) | 4.63 ± 0.44 (40) | 0.757* § |
| **Day 30** | 4.57 ± 0.41 (39) | 4.54 ± 0.35 (40) | 4.61 ± 0.37 (38) | 0.745* § |
| **Day 60**  **End of Study** | 4.55 ± 0.42 (40) | 4.55 ± 0.38 (40) | 4.60 ± 0.39 (37) | 0.779* § |
| **Change from**  **Day 0 to**  **Day 30** | -0.025 ± 0.185 (39) | -0.019 ± 0.127 (40) | -0.001 ± 0.119 (38) | 0.619* Δ |
| **Change from**  **Day 0 to**  **Day 60** | -0.033 ± 0.182 (40) | -0.008 ± 0.152 (40) | -0.009 ± 0.142 (37) | 0.738* Δ |
| **Mean Corpuscular Volume (fL)** | | | | |
| **Day 0**  **Baseline** | 90.3 ± 3.5 (40) | 89.1 ± 3.2 (40) | 90.0 ± 4.5 (40) | 0.318* § |
| **Day 30** | 90.4 ± 3.2 (39) | 89.4 ± 3.3 (40) | 90.1 ± 4.4 (38) | 0.453* § |
| **Day 60**  **End of Study** | 90.2 ± 3.3 (40) | 89.1 ± 3.5 (40) | 89.6 ± 4.4 (37) | 0.409* § |
| **Change from**  **Day 0 to Day 30** | 0.18 ± 1.07 (39) | 0.30 ± 1.92 (40) | 0.05 ± 1.21 (38) | 0.879* Δ |
| **Change from**  **Day 0 to Day 60** | -0.15 ± 1.08 (40) | -0.02 ± 1.89 (40) | -0.54 ± 1.30 (37) | 0.338* Δ |
| **Mean Corpuscular Hemoglobin (pg)** | | | | |
| **Day 0**  **Baseline** | 30.74 ± 1.48 (40) | 30.02 ± 1.29 (40) | 30.34 ± 1.60 (40) | 0.099* § |
| **Day 30** | 30.54 ± 1.30 (39) | 30.08 ± 1.40 (40) | 30.36 ± 1.74 (38) | 0.376* § |
| **Day 60**  **End of Study** | 30.53 ± 1.31 (40) | 29.80 ± 1.40 (40) | 30.42 ± 1.76 (37) | 0.071* § |
| **Change from**  **Day 0 to Day 30** | -0.16 ± 0.73 (39) | 0.06 ± 0.84 (40) | -0.01 ± 0.44 (38) | 0.634* Δ |
| **Change from**  **Day 0 to Day 60** | -0.21 ± 0.70 (40) | -0.22 ± 0.75 (40) | -0.00 ± 0.58 (37) | 0.274* Δ |
| **Mean Corpuscular Hemoglobin Concentration (g/L)** | | | | |
| **Day 0**  **Baseline** | 339.6 ± 6.8 (40) | 337.0 ± 6.1 (40) | 337.4 ± 7.0 (40) | 0.183 § |
| **Day 30** | 338.1 ± 7.1 (39) | 336.8 ± 6.6 (40) | 336.9 ± 7.1 (38) | 0.643 § |
| **Day 60**  **End of Study** | 338.8 ± 7.0 (40) | 334.8 ± 7.1 (40) | 339.2 ± 6.2 (37) | 0.007 § |
| **Change from**  **Day 0 to Day 30** | -1.6 ± 4.9 (39) | -0.2 ± 6.3 (40) | -0.7 ± 4.8 (38) | 0.898 Δ |
| **Change from**  **Day 0 to Day 60** | -0.8 ± 5.2 (40) | -2.3 ± 4.4 (40) | 1.4 ± 6.5 (37) | **0.003** Δ |
| **Red Cell Distribution Width (%)** | | | | |
| **Day 0**  **Baseline** | 13.63 ± 0.56 (40) | 14.00 ± 0.68 (40)^a^ | 13.66 ± 0.46 (40) | **0.008*** § |
| **Day 30** | 13.55 ± 0.54 (39) | 13.98 ± 0.63 (40) | 13.69 ± 0.48 (38) | 0.002* § |
| **Day 60**  **End of Study** | 13.53 ± 0.58 (40) | 13.91 ± 0.63 (40) | 13.66 ± 0.45 (37) | 0.011* § |
| **Change from**  **Day 0 to Day 30** | -0.062 ± 0.298 (39) | -0.015 ± 0.280 (40) | 0.021 ± 0.204 (38) | 0.150* Δ |
| **Change from**  **Day 0 to Day 60** | -0.10 ± 0.37 (40) | -0.08 ± 0.41 (40) | -0.02 ± 0.27 (37) | 0.388* Δ |

S4 Table Continued: Haematology and Clinical Chemistry Parameters of All Randomized Participants at Baseline (Day 0), and Day 30, and Day 60 (N = 120).

|  | **Placebo** | | **Basis 1X** | **Basis 2X** | **Between Group**  **P Value** |
| --- | --- | --- | --- | --- | --- |
|  | **Mean ±SD (n)** | | **Mean ±SD (n)** | **Mean ±SD (n)** |  |
| **Platelet Count (x E9/L)** | | | | | |
| **Day 0**  **Baseline** | 251 ± 47 (40) | | 244 ± 51 (39) | 252 ± 57 (40) | 0.758* § |
| **Day 30** | 250 ± 48 (39) | | 246 ± 51 (40) | 251 ± 59 (38) | 0.933* § |
| **Day 60**  **End of Study** | 250 ± 49 (40) | | 253 ± 54 (39) | 250 ± 60 (37) | 0.942* § |
| **Change from**  **Day 0 to**  **Day 30** | 0.2 ± 20.3 (39) | | 2.5 ± 24.5 (39) | -0.6 ± 25.7 (38) | 0.850* Δ |
| **Change from**  **Day 0 to**  **Day 60** | -0.8 ± 21.6 (40) | | 8.9 ± 28.4 (38) | -1.2 ± 25.4 (37) | 0.134* Δ |
| **Neutrophil Count (x E9/L)** | | | | | |
| **Day 0**  **Baseline** | 3.18 ± 1.23 (40) | | 3.46 ± 1.43 (40) | 3.17 ± 1.04 (40) | 0.738* § |
| **Day 30** | 3.05 ± 1.15 (39) | | 3.19 ± 0.99 (40) | 2.85 ± 0.96 (38) | 0.384* § |
| **Day 60**  **End of Study** | 3.18 ± 1.44 (40) | | 3.22 ± 0.95 (40) | 2.88 ± 1.12 (37) | 0.293* § |
| **Change from**  **Day 0 to**  **Day 30** | -0.15 ± 0.77 (39) | | -0.27 ± 1.17 (40) | -0.32 ± 0.71 (38) | 0.377* Δ |
| **Change from**  **Day 0 to**  **Day 60** | 0.00 ± 0.79 (40) | | -0.24 ± 1.02 (40) | -0.25 ± 0.95 (37) | 0.282* Δ |
| **Lymphocyte Count (x E9/L)** | | | | | |
| **Day 0**  **Baseline** | 1.80 ± 0.57 (40) | | 1.83 ± 0.64 (40) | 1.76 ± 0.43 (40) | 0.962* § |
| **Day 30** | 1.71 ± 0.48 (39) | | 1.88 ± 0.68 (40) | 1.78 ± 0.48 (38) | 0.628* § |
| **Day 60**  **End of Study** | 1.75 ± 0.49 (40) | | 1.84 ± 0.59 (40) | 1.76 ± 0.45 (37) | 0.771* § |
| **Change from**  **Day 0 to Day 30** | -0.082 ± 0.266 (39) | | 0.045 ± 0.292 (40) | 0.032 ± 0.266 (38) | 0.264* Δ |
| **Change from**  **Day 0 to Day 60** | -0.045 ± 0.343 (40) | | 0.012 ± 0.236 (40) | 0.016 ± 0.285 (37) | 0.639* Δ |
| **Monocyte Count (x E9/L)** | | | | | |
| **Day 0**  **Baseline** | 0.497 ± 0.151 (40) | 0.503 ± 0.191 (40) | | 0.473 ± 0.157 (40) | 0.682 † |
| **Day 30** | 0.485 ± 0.137 (39) | 0.477 ± 0.191 (40) | | 0.458 ± 0.120 (38) | 0.785 † |
| **Day 60**  **End of Study** | 0.497 ± 0.202 (40) | 0.500 ± 0.178 (40) | | 0.465 ± 0.148 (37) | 0.729 † |
| **Change from**  **Day 0 to Day 30** | -0.013 ± 0.092 (39) | -0.025 ± 0.150 (40) | | -0.011 ± 0.095 (38) | 0.879 † |
| **Change from**  **Day 0 to Day 60** | 0.000 ± 0.118 (40) | -0.002 ± 0.140 (40) | | -0.014 ± 0.111 (37) | 0.701 † |
| **Eosinophil Count (x E9/L)** | | | | | |
| **Day 0**  **Baseline** | 0.182 ± 0.108 (40) | 0.178 ± 0.103 (40) | | 0.182 ± 0.103 (40) | 0.901 † |
| **Day 30** | 0.179 ± 0.149 (39) | 0.198 ± 0.125 (40) | | 0.192 ± 0.115 (38) | 0.374 † |
| **Day 60**  **End of Study** | 0.180 ± 0.094 (40) | 0.195 ± 0.106 (40) | | 0.189 ± 0.110 (37) | 0.810 † |
| **Change from**  **Day 0 to Day 30** | -0.005 ± 0.102 (39) | 0.020 ± 0.091 (40) | | 0.008 ± 0.063 (38) | 0.110 † |
| **Change from**  **Day 0 to Day 60** | -0.002 ± 0.092 (40) | 0.017 ± 0.081 (40) | | 0.005 ± 0.066 (37) | 0.763 † |

S4 Table Continued: Haematology and Clinical Chemistry Parameters of All Randomized Participants at Baseline (Day 0), and Day 30, and Day 60 (N = 120).

|  | **Placebo** | | **Basis 1X** | **Basis 2X** | **Between Group**  **P Value** |
| --- | --- | --- | --- | --- | --- |
|  | **Mean ±SD (n)** | | **Mean ±SD (n)** | **Mean ±SD (n)** |  |
| **Basophil Count (x E9/L)** | | | | | |
| **Day 0**  **Baseline** | 0.020 ± 0.041 (40) | 0.025 ± 0.044 (40) | | 0.018 ± 0.038 (40) | 0.704 † |
| **Day 30** | 0.028 ± 0.046 (39) | 0.022 ± 0.042 (40) | | 0.021 ± 0.041 (38) | 0.739 † |
| **Day 60**  **End of Study** | 0.025 ± 0.044 (40) 0 (0 – 0.1) | 0.020 ± 0.041 (40) 0 (0 – 0.1) | | 0.022 ± 0.042 (37) | 0.862 † |
| **Change from**  **Day 0 to Day 30** | 0.008 ± 0.042 (39) | -0.002 ± 0.036 (40) | | 0.003 ± 0.028 (38) | 0.447 † |
| **Change from**  **Day 0 to Day 60** | 0.005 ± 0.045 (40) | -0.005 ± 0.032 (40) | | 0.003 ± 0.037 (37) | 0.472 † |
| **Creatinine Concentration (μmol/L)** | | | | | |
| **Day 0**  **Baseline** | 71.3 ± 12.5 (40) | 69.4 ± 16.0 (40) | | 69.2 ± 16.0 (40) | 0.624* § |
| **Day 30** | 70.9 ± 11.6 (40) | 66.7 ± 14.1 (40) | | 68.6 ± 17.2 (39) | 0.269* § |
| **Day 60**  **End of Study** | 70.1 ± 11.2 (40) | 68.0 ± 15.5 (40) | | 67.0 ± 15.1 (37) | 0.421* § |
| **Change from**  **Day 0 to Day 30** | -0.5 ± 6.5 (40) | -2.8 ± 7.8 (40) | | -0.9 ± 5.7 (39) | 0.181* Δ |
| **Change from**  **Day 0 to Day 60** | -1.3 ± 6.1 (40) | -1.5 ± 7.7 (40) | | -2.9 ± 4.9 (37) | 0.353* Δ |
| **Sodium Concentration (mmol/L)** | | | | | |
| **Day 0**  **Baseline** | 140.75 ± 2.59 (40) | 141.45 ± 1.47 (40) | | 141.12 ± 1.86 (40) | 0.306 § |
| **Day 30** | 140.40 ± 2.57 (40) | 141.32 ± 1.89 (40) | | 141.51 ± 2.14 (39) | 0.061 § |
| **Day 60**  **End of Study** | 141.05 ± 3.00 (40) | 141.60 ± 1.89 (40) | | 140.97 ± 2.02 (37) | 0.442 § |
| **Change from**  **Day 0 to Day 30** | -0.35 ± 1.86 (40) | -0.12 ± 2.05 (40) | | 0.36 ± 1.83 (39) | 0.113 Δ |
| **Change from**  **Day 0 to Day 60** | 0.30 ± 3.00 (40) | 0.15 ± 2.01 (40) | | -0.19 ± 2.07 (37) | 0.612 Δ |
| **Potassium Concentration (mmol/L)** | | | | | |
| **Day 0**  **Baseline** | 4.44 ± 0.38 (40) | 4.39 ± 0.32 (40) | | 4.34 ± 0.34 (40) | 0.506* § |
| **Day 30** | 4.42 ± 0.41 (40) | 4.33 ± 0.33 (40) | | 4.32 ± 0.37 (39) | 0.413* § |
| **Day 60**  **End of Study** | 4.36 ± 0.43 (40) | 4.38 ± 0.27 (40) | | 4.29 ± 0.25 (37) | 0.411* § |
| **Change from**  **Day 0 to Day 30** | -0.02 ± 0.43 (40) | -0.06 ± 0.40 (40) | | -0.03 ± 0.35 (39) | 0.588* Δ |
| **Change from**  **Day 0 to Day 60** | -0.08 ± 0.42 (40) | -0.00 ± 0.38 (40) | | -0.07 ± 0.37 (37) | 0.453* Δ |
| **Chloride Concentration (mmol/L)** | | | | | |
| **Day 0**  **Baseline** | 104.8 ± 3.8 (40) | 105.5 ± 2.5 (40) | | 105.2 ± 3.3 (40) | 0.931 † |
| **Day 30** | 104.9 ± 3.4 (40) | 105.6 ± 3.0 (40) | | 106.1 ± 2.6 (39) | 0.344 † |
| **Day 60**  **End of Study** | 105.0 ± 3.7 (40) | 105.6 ± 2.8 (40) | | 106.0 ± 2.6 (37) | 0.497 † |
| **Change from**  **Day 0 to Day 30** | 0.10 ± 2.26 (40) | 0.07 ± 1.79 (40) | | 0.62 ± 2.50 (39) | 0.533 † |
| **Change from**  **Day 0 to Day 60** | 0.25 ± 2.88 (40) | 0.07 ± 1.82 (40) | | 0.62 ± 1.74 (37) | 0.527 † |

S4 Table: Average Fasting Glucose and hs-CRP Concentrations at Baseline (Day 0) and After Supplementation, with Basis at Day 30, and Day 60 for Participants in the ITT Population (N = 118).

|  | **Placebo** | **Basis 1X** | **Basis 2X** | **Between Group**  **P-Value** |
| --- | --- | --- | --- | --- |
|  | **Mean ±SD (n)** | **Mean ±SD (n)** | **Mean ±SD (n)** |  |
| **Fasting Glucose Concentration (mmol/L)** | | | | |
| **Day 0**  **Baseline** | 5.31 ± 0.44 (38) | 5.35 ± 0.62 (39) | 5.23 ± 0.61 (36) | 0.620* § |
| **Day 30** | 5.21 ± 0.54 (40) | 5.27 ± 0.68 (40) | 5.19 ± 0.58 (38) | 0.863* § |
| **Day 60**  **End of Study** | 5.29 ± 0.51 (40) | 5.30 ± 0.63 (40) | 5.32 ± 0.58 (38) | 0.967* § |
| **Change from**  **Day 0 to Day 30** | -0.11 ± 0.40 (38) | -0.06 ± 0.40 (39) | -0.03 ± 0.39 (36) | 0.696* Δ |
| **Change from**  **Day 0 to Day 60** | -0.02 ± 0.34 (38) | -0.03 ± 0.40 (39) | 0.10 ± 0.33 (36) | 0.283* Δ |
| **hsCRP (mg/L)** | | | | |
| **Day 0**  **Baseline** | 2.57 ± 2.53 (38) | 2.92 ± 2.44 (39) | 1.98 ± 3.52 (36) | 0.055* § |
| **Day 30** | 3.1 ± 6.3 (40) | 3.9 ± 10.2 (40) | 2.4 ± 3.6 (38) | 0.821* § |
| **Day 60**  **End of Study** | 2.8 ± 2.7 (40) | 3.2 ± 4.6 (40) | 2.6 ± 3.7 (38) | 0.533* § |
| **Change from**  **Day 0 to Day 30** | 0.6 ± 5.9 (38) | 1.0 ± 9.7 (39) | 0.5 ± 1.5 (36) | 0.122* Δ |
| **Change from**  **Day 0 to Day 60** | 0.30 ± 1.96 (38) | 0.36 ± 4.15 (39) | 0.72 ± 1.54 (36) | 0.214* Δ |
| § Between Basis™ 500 comparison where made using ANOVA.  Δ Between group comparisons were made using ANCOVA adjusting for baseline.  * The logarithmic transformation was required to achieve normality  ^a^ denotes significant difference compared to placebo as assessed by the Tukey-Kramer post-hoc test.  Probability values P≤0.05 are statistically significant. | | | | |
